# Au-WO_3_ Nanocomposite Coatings for Localized Surface Plasmon Resonance Sensing

**DOI:** 10.3390/ma13010246

**Published:** 2020-01-06

**Authors:** Nuno M. Figueiredo, Filipe Vaz, Luís Cunha, Albano Cavaleiro

**Affiliations:** 1 SEG-CEMMPRE–Centre for Mechanical Engineering, Materials and Processes, University of Coimbra, Rua Luís Reis Santos, 3030-788 Coimbra, Portugal; albano.cavaleiro@dem.uc.pt; 2 Center of Physics, University of Minho, Campus de Gualtar, 4710-057 Braga, Portugal; fvaz@fisica.uminho.pt (F.V.); lcunha@fisica.uminho.pt (L.C.); 3 IPN–LED&MAT–Instituto Pedro Nunes, Laboratory for Wear, Testing and Materials, Rua Pedro Nunes, 3030-199 Coimbra, Portugal

**Keywords:** localized surface plasmon resonance (LSPR) sensing, Au-WO_3_ nanocomposites, Au nanoparticles, refractive index sensitivity, optical properties

## Abstract

Localized surface plasmon resonance (LSPR) gas sensors are gaining increasing importance due to their unique tuneable functional properties. Au-WO_3−x_ nanocomposite coatings, in particular, can be outstandingly sensitive to many different gases. However, a proper understanding of their optical properties and the way in which those properties are correlated to their structure/microstructure, is still needed. In this work, Au-WO_3_ nanocomposite coatings, with Au contents between 0–11 atomic percent, were grown using reactive magnetron co-sputtering technique and were characterized concerning their optical response. The precipitation of Au nanoparticles in the oxide matrix was promoted through thermal annealing treatments until 500 °C. Along with the Au nanoparticles’ morphological changes, the annealing treatments stimulated the crystallization of WO_3_, together with the appearance of oxygen-deficient WO_3−x_ phases. Through theoretical simulations, we have related the LSPR effect with the different structural and morphological variations (namely, size and distribution of the nanoparticles and their local environment), which were a function of the Au content and annealing temperature. Our results suggest that local voids were present in the vicinity of the Au nanoparticles, for all temperature range, and that they should be present in a wide variety of Au-WO_3_ nanocomposites. A theoretical study concerning the refractive index sensitivity was carried out in order to predict the optimal coating design parameters for gas sensing experiments.

## 1. Introduction

There is an increasing interest in the deposition and characterization of nanocomposite materials containing metallic nanoparticles due to their wide range of applications in different fields of science such as in chemical/biological sensors [1], nonlinear optics [2], solar cells [2], catalysis/photocatalysis [3], or in medical/antibacterial materials [4]. The very interesting physical and chemical properties of these nanocomposites can be tuned by changing: (i) the composition, size, shape, and concentration of the nanoparticles and (ii) the composition, structure, and microstructure of the dielectric matrix [5,6,7]. Depending on the application, transition metal oxides are frequently used as an embedding matrix material, whereas free electron metals such as Au, Ag, and Cu, which possess surface plasmon resonances in the visible spectrum (and may therefore originate intense colorations), are usually used as the nanoparticle material. The deposition of nanocomposites consisting of metallic nanoparticles embedded in (or dispersed at the surface of) dielectric matrices, has been accomplished with great success by sputtering techniques due to their inherent advantages [1,7,8].

Tungsten trioxide (WO_3_) is a transition metal oxide that exhibits sub-stoichiometric phase transitions and is considered an n-type semiconductor with a wide band gap. It has a high refractive index and shows good corrosion resistance in very strong acid solutions, great optical modulation, good durability, photochromic behaviour, and excellent coloration efficiency [9,10]. The very interesting physical and chemical properties of WO_3_ make this material suitable for a wide range of applications including catalysis [11] and photocatalysis [9], gas sensors [12,13], solar cells [14] and in electrochromic [15], photochromic [10], photoelectrochromic [16], gasochromic [17], and photoelectrochemical [18] devices. WO_3_ is considered as one of the most important gas sensing materials in the world, being sensitive to many different gas species, like O_3_, CO, NO, NH_3_, H_2_S, and CH_4_ [13,19,20]. By incorporating small amounts of Au into WO_3_ or WO_3−x_ the catalytic performance can be increased [20,21] and the electrochromic and electrochemical properties can be changed [22,23]. Additionally, the incorporation of Au nanoparticles into WO_3_ or WO_3−x_ improves the gas detection sensitivity and selectivity [20] and allows gas sensing by localized surface plasmon resonance (LSPR) [24]. LSPR sensors possess a smaller footprint, are more cost-effective and can show higher sensitivity to small variations of the refractive index than the traditional Surface Plasmon Resonance (SPR) metallic thin film sensors [2,25,26,27,28]. Despite the promising idea of using Au-WO_3_ nanocomposites as LSPR sensors, there are few publications on the subject and much still remains to be done concerning the optimization of the deposition techniques, the coatings’ design and the fundamental understanding of both optical properties and gas sensing mechanisms of these coatings. In this article, the optical properties and the refractive index sensitivity of reactive sputtered Au-WO_3_ nanocomposites were evaluated before and after the application of successive thermal annealing treatments up to 500 °C. From the simulation of the optical properties, a new insight was brought concerning the microstructure of the Au-WO_3_ nanocomposites. Based on theoretical simulations of the refractive index sensitivity, we were able to select the best configuration for LSPR gas sensing.

## 2. Materials and Methods

The Au-WO_3_ coatings were grown using reactive magnetron co-sputtering technique on SQ1 quartz substrates. A tungsten target (99.999% purity) together with a 3:2 mixture of oxygen (99.999% purity) and argon (99.999% purity) gases were used in the depositions. The Au content in the coatings was driven by the number of Au strips incrusted in the target’s erosion track (between 0 and 3, each strip having 20 × 3 × 1 mm^3^). The ultimate vacuum pressure was lower than 7 × 10^−4^ Pa and the coatings were deposited at 0.55 Pa. The target-to-substrate distance was maintained in 6 cm and the rotation speed of the substrate holder was 20 rpm. Prior to each deposition, the substrates’ surfaces were cleaned using an electron/ion gun (the procedure consisted of 10 min of electron heating followed by 10 min of Ar^+^ bombardment). A RPG-50 ENI pulsed dc power supply (Rome, Italy) was used with a constant power density of 4 W·cm^−2^, a pulse frequency of 250 kHz and an off-time period of 1456 ns.

After the deposition process, the coatings were subject to thermal annealing treatments at different temperatures (in the 200 °C to 500 °C range) in a horizontal furnace with protective atmosphere (Ar + H_2_ gas mixture). The heating ramp and isothermal period were set to 30 °C/min and 60 min, respectively. The optical transmittance of the coatings was analysed using a Shimadzu UV-3101-PC UV-Vis-NIR spectrophotometer (Kyoto, Japan).

## 3. Results and Discussion

### 3.1. Fundamental Characterization

Four nanocomposite coatings with Au contents up to 11 at.% were deposited and thermally annealed at increasing temperatures up to 500 °C. A prior study of the structural and morphological features of a similar system was published elsewhere [29]. Table 1 resumes the main observations. All as-deposited coatings showed a quasi-amorphous WO_3_ phase, which crystalized into different WO_3−x_ phases with the temperature increase (monoclinic WO_3_ phase at 300–400 °C and orthorombic WO_3_/WO_2.7_ phases at 400–500 °C). The crystallinity of the Au phase increased progressively with both Au content and annealing temperature (the crystallite size varied between ~1.5 nm and ~7 nm).

### 3.2. Optical Properties of Pure WO_3_ Samples

**Figure 1:**
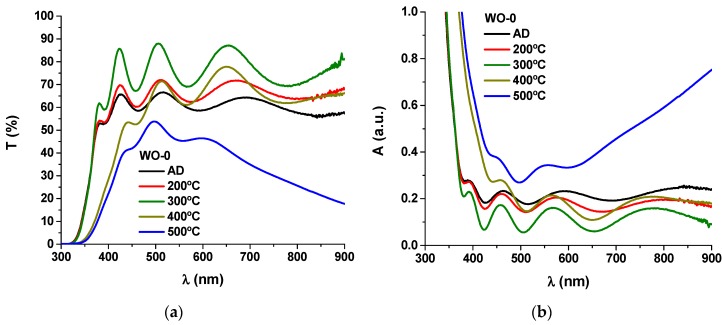
(**a**) Transmittance *T* and (**b**) absorbance *A* as a function of the wavelength *λ* in the visible range for the pure tungsten oxide coatings (sample WO-0) deposited on quartz substrate with the annealing temperature (A.D. stands for as-deposited).

Figure 1a,b shows the transmittance and absorbance plots, respectively, of pure WO_3_ samples before and after each thermal annealing treatment. The optical absorbance (*A*) was obtained using the following relation: *A* = −log *T* [1,30].

The as-deposited sample shows transmittance values close to 65%, in good agreement with the literature [31,32,33,34]. The transmittance increases progressively with the annealing treatments up to 300 °C, reaching values greater than 80%. These changes should be linked to the variation in the atomic packing density of the coating due to the formation of nanocrystalline WO_3_ phases [35]. For higher temperatures, it is observed a damping in the entire visible and near-infrared region, with increasing intensity in the higher wavelength region for the thermal annealing at 500 °C. The transmittance values at 500 °C are close to 40−50% in the 400 nm to 650 nm region and around 20−30% in the 650 nm to 900 nm range. The presence of oxygen deficiencies in the coatings, as attested by the formation of substoichiometric WO_3–x_ phases (Table 1), should be responsible for the increased absorbance in the near-infrared region due to polaron absorption [33,36,37,38,39,40].

**Figure 2:**
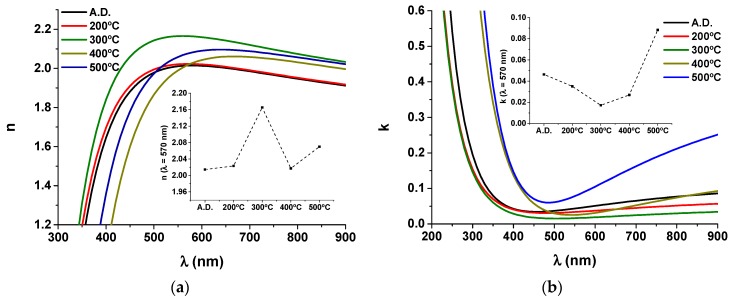
(**a**) Refractive index *n* and (**b**) extinction coefficient *k* as a function of the wavelength *λ* calculated for the pure tungsten oxide(s) coatings, before and after each thermal annealing treatment (A.D. stands for as-deposited). Insets of Figure 2a,b show *n* and *k* values, respectively, calculated at λ = 570 nm.

From the optical transmittance measurements of the pure WO_3_ coatings deposited on quartz substrate (Figure 1a), the refractive index *n* and the extinction coefficient *k* were calculated, as a function of the wavelength *λ*, in the visible and near-infrared region, using Swanepoel’s method [41]. The resulting indices were then fitted to a Cauchy relation of the form $a_{1}+a_{2}/\lambda +a_{3}/\lambda^{4}$. Figure 2a,b shows the calculated refractive index and extinction coefficient dispersion curves, respectively.

Photo-induced refractive index/extinction variations can be a result of structural rearrangements [42]. In this case the refractive index of the pure WO_3_ sample increases progressively with the annealing temperature until 300 °C, and then decreases until 500 °C (Figure 2a), from *n_A.D._* = 2.01 to *n*_300°C_ = 2.16 and finally to *n*_500°C_ = 2.04 (for *λ* = 570 nm). These results are in good agreement with literature; Hutchins et al. [43] have found refractive index values of 2.01 and 2.19 for amorphous and crystalline WO_3_ coatings, respectively. The extinction coefficient displays an opposite trend relative to the refractive index, diminishing with the annealing temperature until 300 °C and then increasing until 500 °C (especially for the higher wavelengths (Figure 2b)), from *k_A.D._* = 0.046 to *k*_300°C_ = 0.017 and finally to *k*_500°C_ = 0.089 (sample value for *λ* = 570 nm). The sample showing the highest refractive index and lowest extinction coefficient—the sample annealed at 300 °C—displays the highest transmittance values in Figure 1a, close to 80%. 

It is of common knowledge that the refractive index is proportional to the density of a coating. Following the Lorentz–Lorenz equation [44]:

(1) $$\frac{n^{2}-1}{n^{2}+2}=\frac{N\alpha_{p}}{3\varepsilon_{0}}\propto \rho$$

where *n*, *α_p_*, *N*, *ρ,* and *ε*_0_ are the refractive index, polarizability of dipoles, number of dipoles per unit volume, coating density, and permittivity of free space, respectively. Assuming a bulk WO_3_ refractive index of 2.5 [45,46], the estimated relative coating densities $\rho /\rho_{0}$ are 0.79, 0.86, and 0.81 for the as-deposited, 300 °C annealed and 500 °C annealed samples, respectively. The increase in the coating refractive index from as-deposited to 300 °C is related to the higher density of the coatings, due to the crystallization of WO_3_ phases, and the reduction in the refractive index observed from 300 °C to 500 °C can be ascribed to the formation of oxygen deficiencies and/or voids in the coatings (higher porosity).

Considering the following relation between the absorption coefficient *α* and the extinction coefficient *k* [30]:

(2) $$\alpha =\frac{4\pi k}{\lambda }.$$

**Figure 3:**
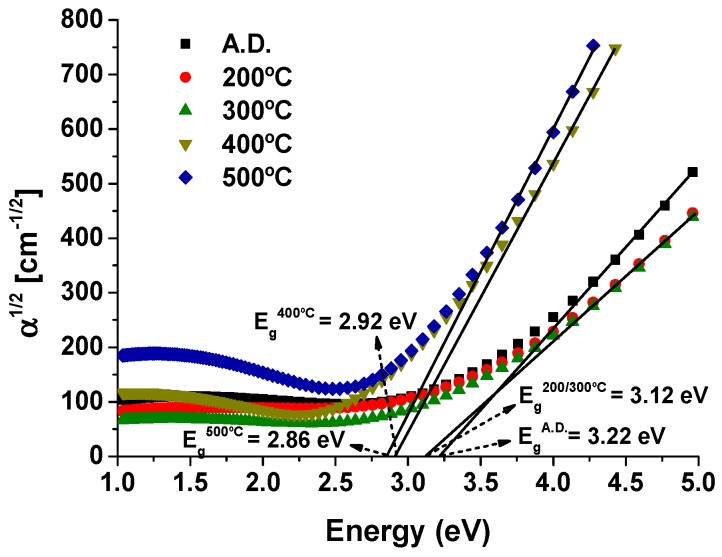
Energy gap extrapolation plots for pure tungsten oxide(s) coatings, before (A.D.) and after thermal annealing at increasing temperatures reaching 500 °C. Indirect allowed transitions were assumed in order to calculate *E_g_* from Bardeen’s equation.

The optical band gap *E_g_* of the pure WO_3_ samples can be determined using the Bardeen equation [47,48]:

(3) $${\left(\alpha h\nu \right)}^{r}=A\left(h\nu -E_{g}\right)$$

where *A* is a parameter that is dependent on the electron-hole mobility, hν is the photon energy and *r* is a characteristic number of the transition process, where *r* = 2 for direct allowed transitions, *r* = 2/3 for direct forbidden transitions, *r* = 1/2 for indirect allowed transitions, and *r* = 1/3 for indirect forbidden transitions. As shown in Figure 3, the ${\left(\alpha h\nu \right)}^{1/2}$ versus hν plot gives a straight line for an energy range over the absorption edge.

The optical band gap was found to be between 3.17 eV (as-deposited and 200 °C annealed samples) and 3.19 eV (300 °C annealed sample) for the quasi-amorphous/nanocrystalline samples, and between 2.78 eV (400 °C annealed sample) and 2.74 eV (500 °C annealed sample) for the crystalline samples. This is in good accordance with reported values of *E_g_*, e.g., 3.18 eV [33], 3.2 eV [49], or 3.22 eV [32,33] for amorphous WO_3_, and 2.7 eV [50,51], 2.8 eV [52], or 2.86 eV [51] for crystalline WO_3_ samples. The reduction in *E_g_* with increasing temperature is mainly caused by the crystallization of WO_3_ phases [51,53] and by the formation of oxygen vacancies [51,54]. The electronic structure of WO_3_ and WO_3−x_ coatings is dependent on their structural properties. The structure of the crystalline material is based on the corner-sharing WO_6_ octahedra; although similar elementary building blocks exist in the amorphous structure, both bond angles and bond lengths display considerable disorder. The valance band and the conduction band consist mainly of O 2p and W 5d orbitals, respectively. Phase transitions modify the W 5d states, thus changing the optical band gap [55,56]. The formation of oxygen vacancies, on the other hand, creates a defect band close to the Fermi level, which is then moved upward, causing the excess electrons to enter the empty lower part of the new defect band, thus decreasing the optical band [51,54,55].

### 3.3. Optical Properties and Refractive Index Sensitivity of Nanocomposite Au-WO_3_ Samples

#### 3.3.1. Experimental Optical Absorbance Spectra

**Figure 4:**
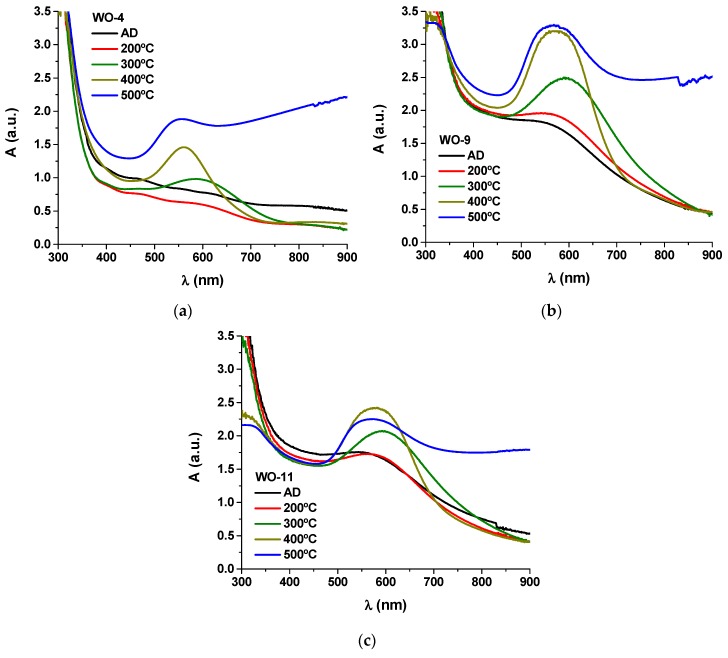
Absorbance *A* as a function of wavelength *λ* in the visible range for the Au-doped WO_3_ samples according to the Au content: (**a**) WO-4, (**b**) WO-9, and (**c**) WO-11.

**Figure 5:**
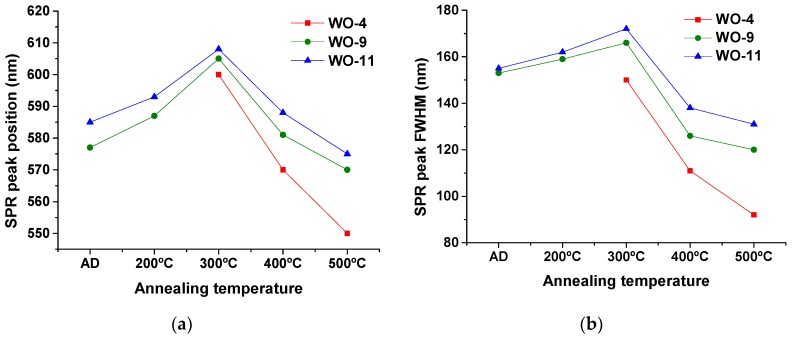
Localized Surface Plasmon Resonance (LSPR) peak (**a**) position (λ_SPR_) and (**b**) full width at half maximum (FWHM).

The absorbance curves of the Au-WO_3_ samples are shown in Figure 4, before and after the different thermal annealing treatments performed up to 500 °C. The colour pictures of the Au-WO_3_ samples can be consulted in Table S1 (Supporting Information). Figure 5 shows the evolution of the SPR peak position and full width at half maximum (FWHM) for the different samples and experimental conditions.

For the as-deposited coatings, the LSPR absorption peaks only appear for Au contents above 9 at.% (Figure 4b,c). The sample with lower Au content (4 at.% Au) only shows LSPR absorption for annealing temperatures higher than 300 °C (Figure 4a). In the overall, the LSPR extinction peaks are significantly enhanced with the annealing temperature (especially for T ≥ 300 °C), becoming more intense and narrow (Figure 5b). All samples annealed at 500 °C show increased absorption at the higher wavelengths originating from the oxygen deficiencies in the tungsten oxide matrix. The intensity increase in the LSPR absorption band with the temperature is mostly caused by the size increase of the Au nanoparticles [28,57]. Additionally, the LSPR peak position varies greatly with the annealing temperature (Figure 5a), red-shifting away from λ_SPR_^AD^ ≈ 580 nm to λ_SPR_^300°C^ ≈ 605 nm and then blue-shifting back to λ_SPR_^500°C^ ≈ 565 nm. For spherical nanoparticles, the variations in the LSPR peak position can be mainly attributed to variations in the refractive index of the host matrix: the LSPR peak red-shifts with increasing refractive index of the host medium owing to the buildup of polarization charges that weakens the total restoring force on the dielectric side of the interface [1,28,57]. As a practical example of how the refractive index of the host medium influences the positioning of the LSPR peaks, for SiO_2_ (*n_host_* ≈ 1.5) λ_SPR_ ≈ 540 nm [58,59], for YSZ (*n_host_* ≈ 2.1) λ_SPR_ ≈ 600 nm [60] and for TiO_2_ (*n_host_* ≈ 2.7) λ_SPR_ ≈ 635 nm [61,62]. Other factors of interest when considering the study of the LSPR peak position are the Au volume fraction and the Au nanoparticle size. By increasing the Au volume fraction the Au interparticle distance decreases and, when it becomes smaller than the nanoparticle diameter, there is an intercoupling of the nanoparticles’ electromagnetic fields that originates a red-shift and a broadening of the LSPR peak [1,28]. Finally, for Au nanoparticles smaller than ~2 nm, two main quantum effects start influencing the plasmon frequency: the electron spill out over the nanoparticle radius and the inner surface-shell of vanishing ionic core polarizability, caused by the localized nature of core-electron wave functions, which can lead to a blue-shift of the LSPR peak [28,57,63]. In the present case, a red-shift up to 300 °C followed by a blue-shift up to 500 °C in the LSPR peak position is qualitatively consistent with the variations observed in the refractive index of the pure tungsten oxides matrix (Figure 2a). However, this variation in *n_h_* (between 2.0–2.16) seems too small to explain the observed changes in the LSPR peak position. In literature, there is some divergence regarding the positioning of the LSPR extinction peaks for coatings of the Au-WO_3_ system. Table 2 shows a set of data taken from several studies on Au-WO_3_ nanocomposites that includes the LSPR peak position (λ_SPR_) and full-width at half maximum (FWHM).

It becomes clear that the SPR peak position varies greatly between 520–610 nm within nanocomposite coatings of the same system. More interestingly yet, is the fact that the SPR peak position of some of the Au-WO_3_ nanocomposites containing embedded Au nanoparticles is similar to the SPR peak position of Au-WO_3_ nanocomposites consisting of Au nanoparticles supported on WO_3_ (λ_SPR_ ≈ 540 nm)—which basically consist of nanoparticles having only 15–30% of their surface area in contact with the matrix (the remaining 70–85% of its surface area is in contact with air). Although never pointed out in any of the cited works, these results clearly suggest that for many Au-WO_3_ nanocomposites consisting of Au nanoparticles embedded in a WO_3_ matrix the local environment of the Au nanoparticles is rich in voids—i.e., at least in the vicinity of the Au nanoparticles the coatings could have increased porosity.

In order to better elucidate the present line of results and further understand the microstructural properties of the Au-WO_3_ coatings, the optical properties of these nanocomposites were simulated using the renormalized Maxwell-Garnett (RMG) effective medium theory, taking into account the nanoparticle size effects. All the theoretical background necessary for performing these simulations is shown in the Appendix A.

#### 3.3.2. Simulation of the Optical Absorbance Spectra

**Figure 6:**
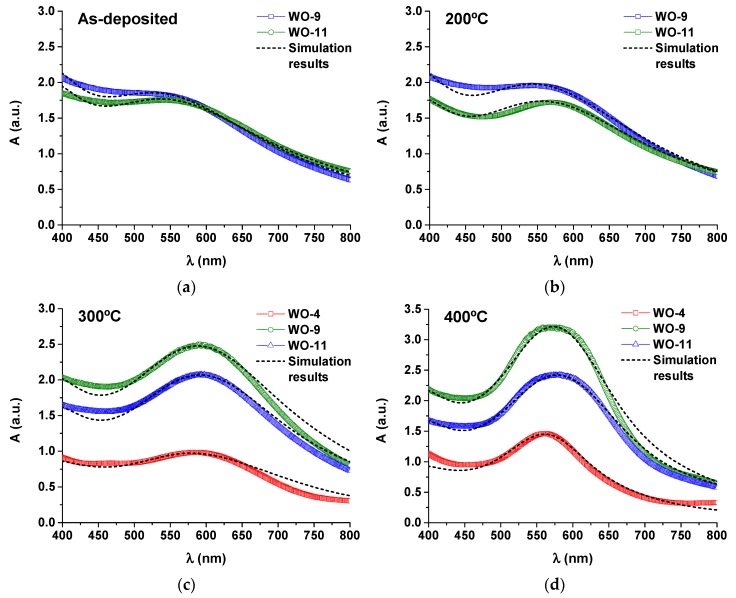

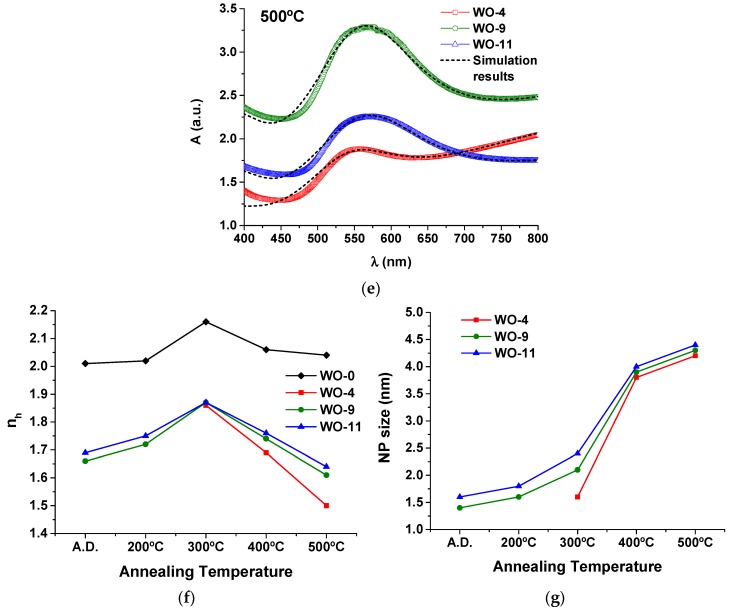
Simulated and experimental absorbance spectra of the Au-WO_3_ samples on quartz substrates with increasing annealing temperature: (**a**) As-deposited, (**b**) 200 °C, (**c**) 300 °C, (**d**) 400 °C, and (**e**) 500 °C. The refractive index of the surrounding medium and the nanoparticle size used in the simulations are depicted in (**f**,**g**), respectively.

The simulations of the absorbance spectra were made considering a thin layer of Au/oxide nanocomposite on a quartz substrate, using the RMG approach for calculating the effective dielectric constant of the medium. For nanoparticle sizes below 2 nm, a two-region core-shell dielectric model was used for the Au nanoparticles, in order to consider the additional quantum confinement effects that are known to originate blue-shifts in the SPR absorption bands. A full description of all the models is given in the Appendix A. To attain a good fit to the experimental data, different parameters were varied: the average diameter of the nanoparticles *d*, the Au volume fraction *f*, the phenomenological parameter *A*, the refractive index of the host medium *n_h_*, and the thickness of the coating *l*. Additionally, at 500 °C, a Lorentzian imaginary part was added to the dielectric function of the tungsten oxide matrix in order to account for the optical extinction at higher wavelengths induced by the oxygen defects in the coatings. Figure 6 shows the simulated absorbance spectra of the Au-containing samples, whereas their resultant fitting parameters are compiled in Table 3. Sample with less Au content (WO-4) there were no LSPR absorption peaks in the as-grown stated and after the 200 °C thermally annealing treatment.

In general terms, the agreement between theory and experiment seems to be quite good.

The simulation results confirm that the increased intensity of the LSPR peaks with the temperature of the annealing treatment was mainly caused by the increase in the nanoparticle size, which varied from ~1.5 nm to ~5 nm (Figure 6g). On the other hand, the overall extinction of the coatings increased with both Au content and thickness, due to the presence of additional absorption/scattering centres. The smaller thickness of sample WO-11 explains its lower absorbance when compared to sample WO-9.

More important are the variations observed in the refractive index of the host medium. We note that the positioning of the LSPR absorption peaks in the visible spectra (Figure 5a) closely follows the trend observed in the refractive index of the host medium (Figure 6d). The refractive index of the host matrix shows a similar trend to one of the pure WO_3_ matrix, by increasing from as-deposited to 300 °C and then decreasing from 300 °C to 500 °C. However, the refractive index of the host medium is at least 0.3 refractive units lower than that of the pure WO_3_ samples, a difference that can only be explained by the presence of voids in the proximity of the Au nanoparticles, i.e., Au seems to induce the formation of voids in the WO_3_ matrix [71]. The formation of voids seems to increase after the thermal annealing at 500 °C. The lower refractive index observed for the samples with lower Au content, annealed at 500 °C, can be partially explained by the lower nanoparticle sizes which, as shown in Table 1, can lead to a higher number of nanoparticles capable of creating more regions with local voids. More generally, our results can help to explain all the discrepancies found in literature concerning the positioning of the LSPR absorption bands. Depending on the size and concentration of Au nanoparticles incorporated in the oxide matrix, a higher (lower) amount of local voids is created, explaining the larger (smaller) blue-shifts in the LSPR absorption bands from the expected LSPR peak positions. We still do not know if there is a direct correlation between the Au nanoparticle size and the tendency of the Au nanoparticle to create local voids (i.e., if changes such as increased reactivity at lower nanoparticle sizes are taking place affecting the formation of voids).

Considering the calculated *n_host_* values, we observe a variation in the coating’s relative density $\rho /\rho_{0}$ from ~0.59 (as-deposited) to ~0.71 (at 300 °C) and to ~0.53 (at 500 °C). On the other hand, the extinction of the host medium increased with both Au content and temperature. Again, this suggests that the incorporation of gold nanoparticles can contribute to the formation of oxygen vacancies and/or voids inside of the W-O matrix. The addition of the Lorentzian term to the imaginary part of *ε_m_* at 500 °C allowed the proper replication of the polaron absorption at the higher wavelengths caused by oxygen vacancies.

#### 3.3.3. Refractive Index Sensitivity of the LSPR Sensors

**Figure 7:**
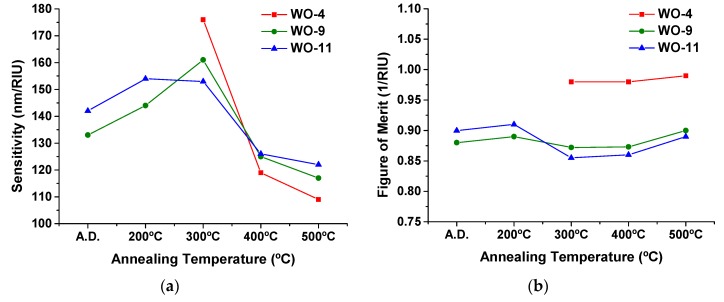
(**a**) Refractive index sensitivity *S* and (**b**) figure of merit FOM calculated for the different Au-WO_3_ samples, considering the simulations presented in Table 3.

The refractive index sensitivity *S* and the figure of merit FOM (for LSPR sensing) of the different Au-WO_3_ coatings were calculated using Equations (A11) and (A12), respectively (both from the Appendix B). The simulation results obtained in the previous section were taken into consideration for these calculations. Figure 7a,b shows the calculated sensitivities and figures of merit, respectively.

The refractive index sensitivity values were generally greater at 300 °C. It was registered an increase with the annealing temperature, from as-deposited up to 300 °C, and above this temperature, up to 500 °C, the refractive index sensitivity decreased progressively. The detected increase of *S*, until *T* = 300 °C, was caused, essentially, by the increase in the host matrix refractive index (see Figure 6d and Equation (A11) of the Appendix B). The reduction in *S*, above *T* = 300 °C, was mainly caused by two factors: (i) a decrease in the plasmon damping constant $\gamma_{P}$ (due to an increasing nanoparticle size—see Equation (A6) in the Appendix A and Equation (A11) in the Appendix B) and (ii) a decrease of the host matrix refractive index with the annealing temperature. The highest *S* value obtained in this study was 176 nm/RIU, for the coating with the lowest Au content (WO-4), after being thermally annealed at 300 °C (RIU means refractive index unit). The sensitivities of samples WO-9 and WO-11 were very similar in the whole range of temperatures, i.e., an increase in the Au content above 9 at.% did not result in a significant improvement of *S*. At 400 °C and 500 °C the sensitivities of samples WO-9 and WO-11 were slightly higher than the one of sample WO-4, mainly due to the lower refractive index of the host matrix observed in the latter case (see Figure 6d).

Concerning the figure of merit (Figure 7b), it generally decreased while increasing the Au content owing to the progressive increase in the FWHM of the LSPR extinction peaks (see Figure 2b and Equation (A12) in the Appendix B). This increase in the FWHM with the Au volume fraction was mostly caused by the increasing interparticle effects (a demonstration of this effect is presented in the last part of Section 3.4). The FOM did not vary greatly with the annealing temperature. The highest FOM value that was obtained in this study was ~1 for the coating with the lowest Au content (WO-4) after the thermal annealing treatment at a temperature equal to or higher than 300 °C.

### 3.4. Theoretical Considerations on the Refractive Index Sensitivity

In this section it is discussed what are the most important coating parameters influencing the refractive index sensitivity, and what is the best choice of parameters to maximize the refractive index sensitivity.

**Figure 8:**
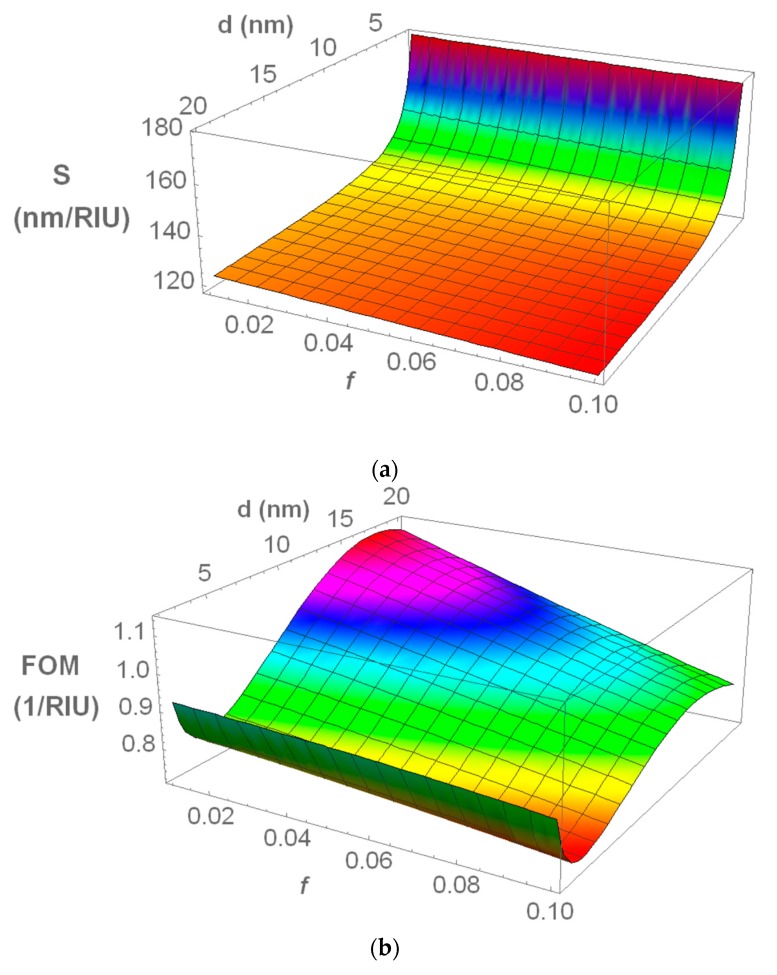
3D surface plot of: (**a**) refractive index sensitivity *S* and (**b**) figure of merit FOM, calculated for a constant *n_h_* = 1.9 and *A* = 1, and for the range of parameters *f* ϵ (0.01–0.1) and *d* ϵ (2–20) nm.

From the refractive index sensitivity expression given in Equation (A11) (in the Appendix B), *S* increases with the host matrix refractive index, $n_{h}$, and with the plasmon damping constant, $\gamma_{P}$, and decreases with the plasma frequency, $\omega_{P}$. However, $n_{h}$, $\gamma_{P}$, $\omega_{P}$, and also *f* and *d*, all influence the SPR peak position $\lambda_{\mathrm{SPR}}$—and, although the impact of $\lambda_{\mathrm{SPR}}$ on *S* is not clear to understand from Equation (A11), from numerical analysis it is observed that *S* also decreases with $\lambda_{\mathrm{SPR}}$. Additionally, $\gamma_{P}$ is a function of the nanoparticle diameter *d* and of the phenomenological parameter *A* (according to Equation (A6)), whereas the plasma frequency $\omega_{P}$ is dependent on the metallic material (which in this case does not vary). It can thus be considered that there are four main parameters influencing the sensitivity of an LSPR sensor: $n_{h}$, *A*, *d*, and *f*. Based on these considerations, by setting a fixed value for $n_{h}=1.9$ and A=1 we can then make a three-dimensional analysis considering the combined effect of the nanoparticle diameter *d* and volume fraction *f* on the refractive index sensitivity *S* and figure of merit FOM of the sensor. From this analysis, we can identify the best possible design parameters for the nanocomposite LSPR sensor containing spherical Au inclusions. The following procedure was applied in this analysis: (i) simulating the nanocomposites’ absorbance spectra for different nanoparticle sizes, *d* (varying between 2 and 20 nm), and metal volume fractions, *f* (varying between 0.01 and 0.1); (ii) extracting the SPR peak parameters (peak position, $\lambda_{\mathrm{SPR}}$, and FWHM); (iii) fitting linear functions of $\lambda_{\mathrm{SPR}}$ and FWHM to these sets of data; (iv) using these functions in the *S* and FOM expressions; and (v) generating three-dimensional surface plots based on these new expressions. Figure 8a,b shows the refractive index sensitivity and figure of merit, respectively, obtained from the abovementioned surface analysis procedure.

It becomes clear that the refractive index sensitivity decreases slightly while increasing the Au volume fraction (mostly due to the increase in $\lambda_{\mathrm{SPR}}$) and that it decreases at different rates, while increasing the nanoparticle size (mostly due to a decrease in $\gamma_{P}$). The impact of the nanoparticle diameter on *S* is more significant at very small sizes (*d* < 3 nm), cases where the plasmon damping constant $\gamma_{P}$ suffers a remarkable increase. A maximum *S* value of 180 nm/RIU was obtained for a 2 nm nanoparticle diameter, whereas for sizes above ~4 nm, *S* shows values of around ~125 nm/RIU.

**Figure 9:**
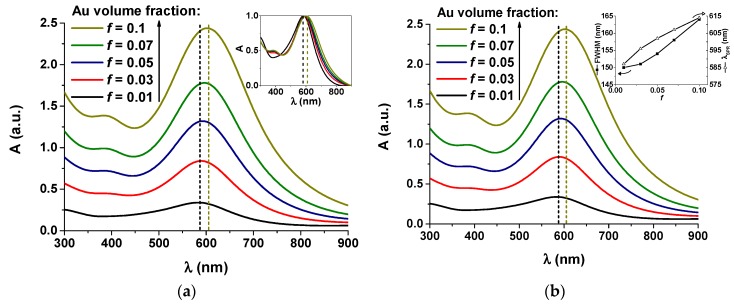
Simulated absorbance spectra of nanocomposite coatings containing spherical Au nanoparticles supported on quartz substrates calculated using: (**a**) a constant *d* of 5 nm and a varying *f* between 0.01 and 0.1. Inset shows the normalized absorbance curves; (**b**) a varying *d* between 2 and 20 nm and a constant *f* of 0.05. Inset shows the evolution of the FWHM. In both cases, the following parameters were used in the simulations: *A* = 1, *n_h_* = 1.9, and *l* = 200 nm.

In the overall, the figure of merit decreases with increasing Au volume fraction due to the progressive increase in the width and in the wavelength position of the LSPR extinction peak. This general behaviour is depicted in Figure 9a. The optimum range of *f* lies between 0.01 and 0.05. Changing the nanoparticle size affects the figure of merit by changing the plasmon damping constant, $\gamma_{P}$, which changes *S* and FWHM in the same direction, but at different rates. Depending on the *S* and FWHM rate of increase/decrease, two distinct regions appear: (i) for very small nanoparticles (*d* < 3 nm), when decreasing the nanoparticle size, *S* increases at a much higher rate than FWHM, resulting in increased values for FOM; (ii) for the case of larger nanoparticles (*d* > 4 nm), while increasing the nanoparticle size, *S* decreases at a lower rate than FWHM until a certain nanoparticle size is reached, increasing the FOM. This general behaviour is depicted in Figure 9b. Thus, there are two ideal regions for the Au nanoparticle size: (i) between 2.0 and 2.5 nm (a maximum FOM of 1.0 was obtained for a nanoparticle size of 2 nm and an *f* of 0.01) and (ii) between 12 and 22 nm (a maximum FOM of 1.1 was obtained for a nanoparticle size of 17 nm and an *f* of 0.01).

## 4. Conclusions

In this study, the optical properties of Au-WO_3_ nanocomposites, having Au contents between 0 and 11 atom % Au, were studied in great detail and related with their refractive index sensitivity. Thermal annealing treatments, carried between 200 °C and 500 °C, promoted the crystallization of WO_3_ phases and the growth of Au nanoparticles, resulting in the enhancement of the LSPR extinction peaks. However, the LSPR peak positions varied greatly with the annealing temperature. A red-shift in the LSPR peak positions from as-deposited (~580 nm) to 300 °C (~605 nm) was observed due to the crystallization of WO_3_ phases, and a strong blue-shift from 300 °C (~605 nm) to 500 °C (~565 nm) was observed due to the formation of oxygen deficiencies and voids in the tungsten oxide matrix. Au seems to induce the formation of oxygen vacancies and/or local voids inside the W-O matrix. The variations in the LSPR extinction peaks with the Au content and annealing temperature allowed to achieve: (i) different refractive index sensitivities, between 110 and 180 nm/RIU, with the best result being found for the nanocomposite with 4 at.% Au after the thermal annealing at 300 °C; (ii) different colour tones (brown, blue, and purple), which can allow this system to be considered as an innovative alternative for decorative applications as well.

A theoretical study of the refractive index sensitivity was performed for nanocomposite coatings having different Au contents and Au nanoparticle sizes. The nanoparticle diameter influenced substantially the refractive index sensitivity for very small sizes (*d* < 3 nm), cases where the plasmon damping constant was significantly increased. In contrast, by increasing the gold volume fraction the refractive index sensitivity was slightly reduced due to the increased dipole–dipole interaction between the nanoparticles, which promoted a broadening and also a red-shift of the LSPR peaks. The best figure of merit values calculated in this study were found for nanocomposites containing 17 nm Au nanoparticles and Au volume fraction values lower than 0.05.

## Figures and Tables

**Table 1:** Representative microstructure of the Au-WO_3_ samples, simulated in Mathematica, using 20 nm × 20 nm × 7 nm unit cells. The Au nanoparticle size and the crystallinity of the matrix are shown as well.

Sample Reference	Sample’s Representative Microstructure (Including Crystallinity of the Matrix)		
	As-Deposited	300 °C	500 °C
**WO-0** **(0 atom % Au)**	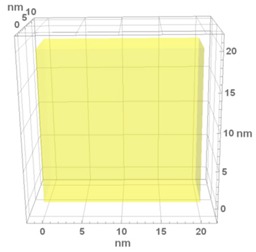 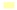 Quasi-amorphous WO_3_	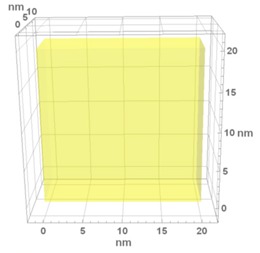 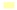 Nanocrystalline WO_3_	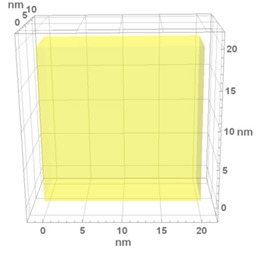 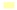 Orthorhombic WO_3_ Orthorhombic WO_2.7_
**WO-4** **(4.0 atom % Au)**	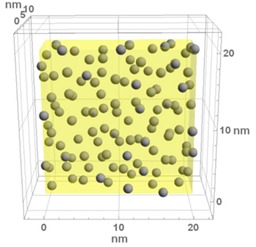 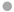 Au NPs size ≈ 1.3 nm 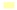 Quasi-amorphous WO_3_	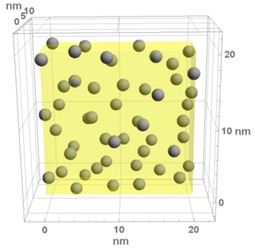 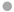 Au NPs size ≈ 1.7 nm 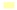 Nanocrystalline WO_3_	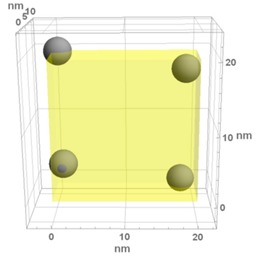 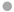 Au NPs size ≈ 4.0 nm 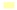 Orthorhombic WO_3_ Orthorhombic WO_2.7_
**WO-9** **(8.8 atom % Au)**	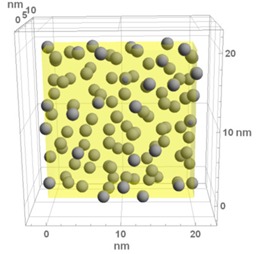 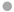 Au NPs size ≈ 1.7 nm 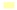 Quasi-amorphous WO_3_	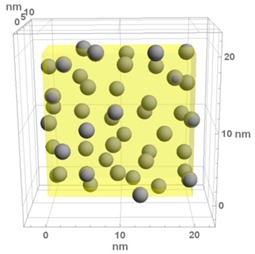 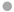 Au NPs size ≈ 2.2 nm 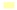 Nanocrystalline WO_3_	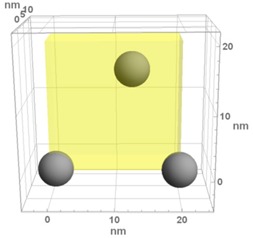 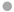 Au NPs size ≈ 5.5 nm 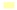 Orthorhombic WO_3_ Orthorhombic WO_2.7_
**WO-11** **(10.6 atom % Au)**	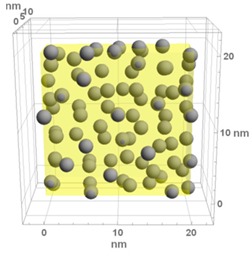 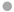 Au NPs size ≈ 2.0 nm 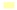 Quasi-amorphous WO_3_	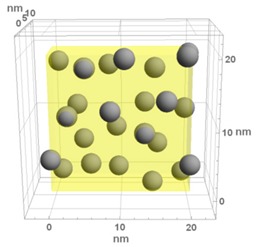 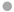 Au NPs size ≈ 3.0 nm 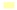 Monoclinic WO_3_	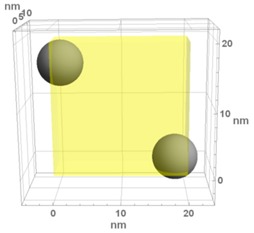 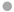 Au NPs size ≈ 7.0 nm 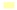 Orthorhombic WO_3_ Orthorhombic WO_2.7_

**Table 2:** LSPR peak position (λ_SPR_), full-width at half maximum (FWHM) and, whenever available, Au nanoparticle size and crystal structure of the W–O phase of several Au-WO_3_ nanocomposites, extracted from literature [22,64,65,66,67,68,69,70].

Type of Nanocomposite	λ_SPR_ (nm)	FWHM (nm)	Au NP Size (nm)	Structure of WO_3_	Reference
Au NPs embedded in WO_3_ films	520	110	-	Amorphous	[64]
	525	100	5	Crystalline	[65]
	550	80	10	Crystalline	[66]
	550	100	-	Amorphous	[67]
	560	100	-	Amorphous	[64]
	579	120	-	-	[68]
	583	253	4	Crystalline	[22]
	604	126	-	Crystalline	[69]
	608	130	-	Crystalline	[69]
Au NPs supported on WO_3_ nanorods	540	80	10	Crystalline	[69]
Au NPs supported on WO_3_ nanoplates	540	244	45	Crystalline	[70]

**Table 3:** LSPR peak position (λ_SPR_), set of parameters used for simulating the absorbance spectra shown in Figure 6, namely the nanoparticle diameter (*d*), the size effect parameter (*A*), the Au volume fraction (*f*), the complex refractive index of the host medium (${\tilde{n}}_{h}=n+i\;k$) and the layer thickness (*l*). A.D. means as-deposited. A core-shell model with a shell thickness of 1–2 a.u. shell of reduced polarizability was used for Au nanoparticles with sizes bellow 2 nm.

Sample	*T* (°C)	*d* (nm)	A	f	${\tilde{n}}_{h}$ (*λ* = 570 nm)	*l* (nm)
WO-4	300	1.6	0.80	0.031	1.86	430
	400	3.8	0.70	0.030	1.69 + 0.005 i	446
	500	4.2	0.75	0.028	1.50 + 0.13 i	454
WO-9	A.D.	1.4	1	0.075	1.66	413
	200	1.6	1	0.076	1.72	411
	300	2.1	0.95	0.077	1.87	414
	400	3.9	0.95	0.076	1.74 + 0.007 i	437
	500	4.3	1.00	0.073	1.61 + 0.12 i	444
WO-11	A.D.	1.6	1	0.97	1.69	290
	200	1.8	1	0.093	1.75	274
	300	2.4	0.95	0.091	1.87	270
	400	4.0	1.00	0.089	1.76 + 0.01 i	280
	500	4.4	1.10	0.079	1.64 + 0.12 i	289

## Supplementary Materials

The following are available online at https://www.mdpi.com/1996-1944/13/1/246/s1, Table S1: Colour pictures of the coatings deposited on quartz substrate in transmittance mode, after the different annealing treatments. As-deposited coatings (not shown) are similar in coloration to the ones annealed at 200 °C.

## Appendix A. Optical Properties of Nanocomposite Coatings

Here it is provided the mathematical treatments used for determining the optical properties of nanocomposites consisting of spherical metal inclusions randomly distributed on a host dielectric matrix.

In the classical Maxwell–Garnett (MG) theory, considering the mean field approximation, the effective dielectric function *ε_eff_* of a nanocomposite coating consisting of a small volume fraction *f* of separate spherical inclusions dispersed on a host dielectric medium is given by [57]:

(A1) $$\frac{\varepsilon_{eff}-\varepsilon_{h}}{\varepsilon_{eff}+2\varepsilon_{h}}=\frac{4\pi }{3}N\bar{\alpha }$$

where *ε_h_* is the host medium dielectric function, $\bar{\alpha }=\sum_{i}\alpha_{i}/3$ is the average nanoparticle polarizability (where $\alpha_{i}$ is the polarizability of the individual particle following a principal axis), and N=f/V is the nanoparticle concentration (*V* is the nanoparticle volume). The polarizability of a single spherical nanoparticle, with radius R≪λ, following a principal axis is given by:

(A2) $$\alpha_{i}=R^{3}\frac{\varepsilon_{m}-\varepsilon_{h}}{\varepsilon_{m}+2\varepsilon_{h}}$$

where *ε_m_* is the metallic inclusions’ dielectric function. Thus, for spherical nanoparticles ($\bar{\alpha }=\alpha_{i}$), Equation (A1) turns into the more familiar expression of *ε_eff_*:

(A3) $$\frac{\varepsilon_{eff}-\varepsilon_{h}}{\varepsilon_{eff}+2\varepsilon_{h}}=f\frac{\varepsilon_{m}-\varepsilon_{h}}{\varepsilon_{m}+2\varepsilon_{h}}.$$

The MG equation is valid for low concentrations (*f* << 1) but can be extended to higher *f* by considering the dipole–dipole interactions between nanoparticles [72], a formalism that is known as the Renormalized Maxwell-Garnett (RMG) approximation [73]. The dipole–dipole interaction among particles renormalizes their average polarizability, which then becomes [72]:

(A4) $$\alpha^{*}=\frac{2\bar{\alpha }}{\kappa }\left[1-\frac{\sqrt{1-\kappa \left(1-\delta \right)}}{2}\left(\sqrt{1-\nu }+\frac{\arcsin\;\nu^{1/2}}{\nu^{1/2}}\right)\right]$$

where ν=3κδ/[1−κ(1−δ)], $\kappa =f{\left(4\pi \bar{\alpha }/3V\right)}^{2}$ and $\delta =\left(\alpha_{⊥}-\alpha_{∥}\right)/\left(\alpha_{⊥}+\alpha_{∥}\right)$ is an anisotropy parameter (for spherical particles δ becomes 0). Note that $\alpha^{*}\to \bar{\alpha }$ when f→0.

In order to represent *ε_m_*, an analytical expression based on the traditional Drude–Lorentz (DL) model was used:

(A5) $$\varepsilon_{m}\left(\omega \right)=1\underset{\begin{array}{c} \text{Drude susceptibility}\\ \chi^{2}(\text{free electrons}) \end{array}}{\underbrace{-\frac{\omega_{P}^{2}}{\omega^{2}+i\gamma_{P}\omega }}}+\underset{\begin{array}{c} \text{Lorentz susceptibility}\\ \chi^{b}(\text{bound electrons}) \end{array}}{\underbrace{\overset{z}{\underset{j=1}{\sum }}\frac{f_{j}\omega_{j}^{2}}{\omega_{j}^{2}-\omega^{2}-i\gamma_{j}\omega }}}$$

where $\omega_{P}$ and $\gamma_{P}$ are the plasma frequency and the damping constant associated with intraband transitions and *z* is the number of oscillators describing the interband transitions, each with resonant frequency $\omega_{j}$, bandwidth $\gamma_{j}$, and strength $f_{j}$. The fitting parameters from reference [74] were used in this work.

The nanoparticles are however sufficiently small so that the mean free path of the conduction electrons *l* should be affected by boundary scattering [75]. These intrinsic size effects were accounted in the DL model by correcting the plasmon damping parameter $\gamma_{P}$ according to the equation [57]:

(A6) $$\gamma_{P}=\gamma_{0}+\frac{A\;\upsilon_{F}}{R}$$

where $\gamma_{0}$ is the bulk metal relaxation constant, *A* is a parameter that is a function of the nanoparticle geometry, of the order of unity, *ν_F_* is the Au Fermi velocity (1.39 × 10^15^ nm/s) and *R* is the nanoparticle radius (in nm).

For Au nanoparticle sizes below 2 nm [57], an additional quantum effect comes into play: because of the localized nature of core-electron wave functions, the screening effects become less effective above a surface layer within the metal nanoparticle, originating a blue-shift of the SPR peak. This effect can be taken into consideration in a two-region core-shell dielectric model where the effective polarizable continuous medium that is responsible for the screening does not extend over the entire nanoparticle volume, i.e., by making $\chi^{b}$ to vanish (from Equation (A5)) for a radius greater than $R-d_{shell}$, where *R* is the nanoparticle radius and *d_shell_* is the shell thickness (*d_shel_*_l_ ≈ 1–2 atomic units ≈ 0.05–0.11 nm) [76]. The polarizability of a core-shell nanoparticle is given by [77]:

(A7) $$\alpha_{i}=R^{3}\frac{\left(\varepsilon_{s}-\varepsilon_{h}\right)\left(\varepsilon_{c}+2\varepsilon_{s}\right)+g\left(\varepsilon_{c}-\varepsilon_{s}\right)\left(\varepsilon_{h}+2\varepsilon_{s}\right)}{\left(\varepsilon_{s}+2\varepsilon_{h}\right)\left(\varepsilon_{c}+2\varepsilon_{s}\right)+g\left(2\varepsilon_{s}-2\varepsilon_{h}\right)\left(\varepsilon_{c}-\varepsilon_{s}\right)}$$

where $\varepsilon_{s}=1-\chi^{f}$ is the dielectric function of the shell, $\varepsilon_{c}\equiv \varepsilon_{m}=1-\chi^{f}-\chi^{b}$ is the core dielectric function, *ε_h_* is the host medium dielectric function and $g={\left(R-d_{shell}\right)}^{3}/R^{3}$ is the volume fraction of the inner sphere (concerning the total nanoparticle volume). Thus, for nanoparticle sizes bellow 2 nm, Equation (A7) should be used (instead of Equation (A2)) in Equation (A1) (MG) or in Equation (A4) (RMG). To our best knowledge, this study is the first one suggesting the latter strategy, i.e., to combine the core-shell nanoparticle model, for simulating the blue-shifts that occur in very small Au nanoparticles, and the renormalized polarizability, for simulating the red-shifts occurring for Au volume fractions above ~5%.

After having determined the effective dielectric constant of the medium (*ε_eff_*), the optical transmittance (*T*) of the nanocomposites can be calculated at normal incidence using the transfer matrix method [78,79]. The absorbance (*A*) can be determined through the relation [80]: *A* = −log *T*.

## Appendix B. Refractive Index Sensitivity of LSPR Sensors

Two main strategies are commonly employed for quantifying variations in the LSPR peak: (i) by determining the variation in the positioning of the peak maxima or (ii) by calculating the difference in intensity at a suitable point in the peak. In this work the former strategy was used.

The refractive index sensitivity of an LSPR sensor with spectral interrogation is defined as the ratio of resonant wavelength shift $d\lambda_{\mathrm{SPR}}$ to the variation of surrounding refractive index $dn_{h}$ [1,81,82,83,84]:

(A8) $$S=\frac{d\lambda_{\mathrm{SPR}}}{dn_{h}}=\frac{{\left(\frac{d\varepsilon_{1}\left(\lambda \right)}{dn_{h}}\right)}_{\lambda =\lambda_{\mathrm{SPR}}}}{{\left(\frac{d\varepsilon_{1}\left(\lambda \right)}{d\lambda }\right)}_{\lambda =\lambda_{\mathrm{SPR}}}}\;\left(\mathrm{nm}/\mathrm{RIU}\right)$$

where $\varepsilon_{1}\left(\lambda \right)$ is the real part of the complex metal dielectric function $\varepsilon_{m}\left(\lambda \right)=\varepsilon_{1}\left(\lambda \right)+i\varepsilon_{2}\left(\lambda \right)$. From Equation (A5), and by using the equality ω=2πc/λ (where *c* is the speed of light in free space), the following expression for $\varepsilon_{1}\left(\lambda \right)$ is obtained:

(A9) ε1(λ)=1+Re[χb(2πcλ)]−ωp2(2πcλ)2+γp2.

The refractive index sensitivity of the resonance is determined by the same two relations determining the resonance wavelength $\lambda_{\mathrm{SPR}}$: the wavelength-dependent dielectric function and the resonance condition [81].

Resonance occurs at poles of the polarizability. At wavelengths where the imaginary part of the metal dielectric function is small or slowly varying, the pole of polarizability is obtained by equalling the real part of the denominator in Equation (A2) to zero. This results in the following resonance condition [81]:

(A10) $$\varepsilon_{1}\left(\lambda_{\mathrm{SPR}}\right)=-2n_{h}^{2}$$

which determines the real part of the dielectric function at resonance.

Substituting Equations (A9) and (A10) into Equation (A8) results in the following expression for the LSPR refractive index sensitivity [1]:

(A11) $$S=\frac{2n_{h}\lambda_{\mathrm{SPR}}{\left[{\left(\frac{2\pi c}{\lambda_{\mathrm{SPR}}}\right)}^{2}+\gamma_{P}^{2}\right]}^{2}}{{\left(\frac{2\pi c}{\lambda_{\mathrm{SPR}}}\right)}^{2}\omega_{P}^{2}}\;\left(\mathrm{nm}/\mathrm{RIU}\right).$$

The ability of a spectroscopic SPR sensor to resolve small refractive index changes is directly proportional to the refractive index sensitivity and indirectly proportional to the width of the resonant feature. Thus, to further deduce a coating’s sensing potential, the following figure of merit (FOM) can be used [1,82,83,85]:

(A12) $$\mathrm{FOM}=\frac{S}{{\mathrm{FWHM}}_{\mathrm{SPR}}}\;\left({\mathrm{RIU}}^{-1}\right)$$

where *S* is the refractive index sensitivity and ${\mathrm{FWHM}}_{\mathrm{SPR}}$ is the full width at half maximum of the LSPR extinction peak.
