# Supplementary material for: Au-WO3 Nanocomposite Coatings for Localized Surface Plasmon Resonance Sensing
Source: Materials (Basel). 2020 Jan 6;13(1):246. doi: 10.3390/ma13010246 (PMC6981964; doi:10.3390/ma13010246)
Supplement: Supplementary file 1 [file materials-13-00246-s001.pdf]

materials

Supplementary Materials: Au-WO_3 Nanocomposite Coatings for Localized Surface Plasmon Resonance Sensing

Nuno M. Figueiredo [1,*], Filipe Vaz [2], Luís Cunha [2] and Albano Cavaleiro [1,3]

[1] SEG-CEMMPRE–Centre for Mechanical Engineering, Materials and Processes, University of Coimbra, Rua Luís Reis Santos, 3030-788 Coimbra, Portugal; albano.cavaleiro@dem.uc.pt
[2] Center of Physics, University of Minho, Campus de Gualtar, 4710-057 Braga, Portugal; fvaz@fisica.uminho.pt (F.V.); lcunha@fisica.uminho.pt (L.C.)
[3] IPN–LED&MAT–Instituto Pedro Nunes, Laboratory for Wear, Testing and Materials, Rua Pedro Nunes, 3030-199 Coimbra, Portugal
* Correspondence: nuno.figueiredo@dem.uc.pt; Tel.: +351-239790745

Table S1. Colour pictures of the coatings deposited on quartz substrate in transmittance mode, after the different annealing treatments. As-deposited coatings (not shown) are similar in coloration to the ones annealed at 200 °C.

Sample Reference	Annealing Temperature (°C)			
	200	300	400	500
WO-0				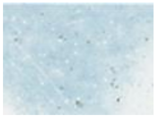
WO-4	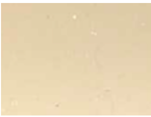	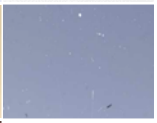	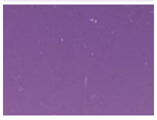	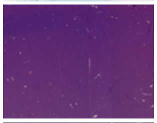
WO-9	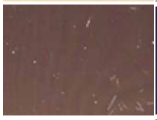	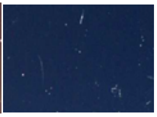	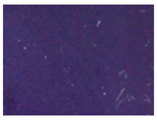	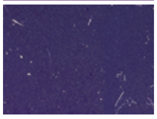
WO-11	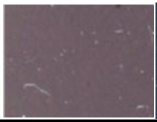	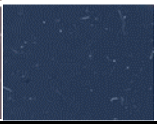	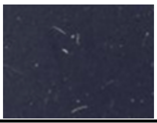	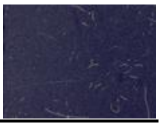

Table S1 comment: The pure WO_3 sample shows no coloration up to 400 °C but presents light bluish tones at 500 °C. This colour has been ascribed to WO_3 coatings having oxygen deficiencies [37–39]. Regarding the Au-containing samples, they show: (i) different tones of brown colour at 200 °C (cases where the LSPR peaks are wide and very low intense, generating continuous absorption bands in the ~350–600 nm range), (ii) different tones of blue colour at 300 °C (cases where the LSPR peaks are red-shifted, allowing more blue light and less yellow/red light to be transmitted) and (iii) different tones of purple/blue/dark blue colour at 400–500 °C with the increasing Au content, respectively (being this related to the blue-shift and enhancement of the LSPR peaks and also to the polaron absorption in the higher wavelengths). Because of the highly tuneable electrical and optical properties of the tungsten oxide matrix, this Au-WO_3 system could be used as innovative decorative coatings as well. In fact, this system can be a good alternative in the decorative coatings industry, since a large palette of colors can be achieved with only one single deposition system of elements, a very attractive procedure for industrial production.
